# Additively manufactured controlled porous orthopedic joint replacement designs to reduce bone stress shielding: a systematic review

**DOI:** 10.1186/s13018-022-03492-9

**Published:** 2023-01-16

**Authors:** Sarah Safavi, Yihang Yu, Dale L. Robinson, Hans A. Gray, David C. Ackland, Peter V. S. Lee

**Affiliations:** Department of Biomedical Engineering, University of Melbourne, Parkville, VIC 3010 Australia

**Keywords:** 3D printing, Lattice structure, Aseptic loosening, Joint prosthesis, Orthopedic implant, Osseointegration

## Abstract

**Background:**

Total joint replacements are an established treatment for patients suffering from reduced mobility and pain due to severe joint damage. Aseptic loosening due to stress shielding is currently one of the main reasons for revision surgery. As this phenomenon is related to a mismatch in mechanical properties between implant and bone, stiffness reduction of implants has been of major interest in new implant designs. Facilitated by modern additive manufacturing technologies, the introduction of porosity into implant materials has been shown to enable significant stiffness reduction; however, whether these devices mitigate stress-shielding associated complications or device failure remains poorly understood.

**Methods:**

In this systematic review, a broad literature search was conducted in six databases (Scopus, Web of Science, Medline, Embase, Compendex, and Inspec) aiming to identify current design approaches to target stress shielding through controlled porous structures. The search keywords included ‘lattice,’ ‘implant,’ ‘additive manufacturing,’ and ‘stress shielding.’

**Results:**

After the screening of 2530 articles, a total of 46 studies were included in this review. Studies focusing on hip, knee, and shoulder replacements were found. Three porous design strategies were identified, specifically uniform, graded, and optimized designs. The latter included personalized design approaches targeting stress shielding based on patient-specific data. All studies reported a reduction of stress shielding achieved by the presented design.

**Conclusion:**

Not all studies used quantitative measures to describe the improvements, and the main stress shielding measures chosen varied between studies. However, due to the nature of the optimization approaches, optimized designs were found to be the most promising. Besides the stiffness reduction, other factors such as mechanical strength can be considered in the design on a patient-specific level. While it was found that controlled porous designs are overall promising to reduce stress shielding, further research and clinical evidence are needed to determine the most superior design approach for total joint replacement implants.

## Introduction

Total knee replacement (TKR) and total hip replacement (THR) surgeries are established treatments to improve quality of life by reducing pain and restoring mobility in patients suffering from advanced osteoarthritis [1, 2]. By 2030, the annual number of primary TKR and THR procedures in the USA is projected to be 2.8–4.1 million [3]. In Australia, over 1.8 million joint replacement surgeries were reported between 1999 and 2021 with the number of TKR and THR surgeries expected to increase by over 200% in the next 30 years [4, 5]. The number of total shoulder replacements (TSR) has been increasing substantially and is projected to be up to over 200% higher in 2025 compared to 2017 [4, 6, 7]. Current revision rates are approximately 6% after five years and 12% after ten years for both THR and TKR arthroplasties, respectively [8]. These high revision rates are compounded by the increasing number of young patients undergoing hip and knee joint replacement surgery [9]. Together these factors underscore the critical importance of longevity in modern implant designs.

The leading cause for implant loosening and periprosthetic fractures is loss of bone density due to stress shielding [10, 11]. A key factor that influences stress shielding is the mismatch in the material properties between the implant and bone. The elastic modulus of common implant materials such as titanium and its alloys is within 100–120 GPa, while the elastic modulus of bone is within 0.02–6 GPa for cancellous and 3–30 GPa for cortical bone [12]. Due to the significantly higher stiffness of the implant, the load is primarily transferred through the implant which shields the bone from loading and results in bone resorption as per Wolff’s law [11, 13].

Several design approaches have been adopted in recent years with the aim to reduce implant stiffness and mitigate stress shielding. These include alterations of the implant geometry [10] and the use of low-stiffness materials [10]. Another approach to reduce stiffness while additionally enhancing implant fixation is the introduction of empty spaces into the implant design, creating porosity. Implant porosity enhances the fixation to the bone as it enables osseointegration which is the ingrowth of bone into the implant [14]. Osseointegration requires low relative micromotion between the implant and the bone (< 150 μm) which can be achieved through reduced implant stiffness and thus reduced stresses at the bone–implant interface [14, 15]. Porous materials are therefore advantageous for increasing implant longevity compared to other design approaches.

Additive manufacturing (AM) is an emerging technology in the medical device sector, allowing for more flexibility in the internal design structure of implants. At present, AM has been used to fabricate implants for a number of locations in the body including the spine, the hip, and maxillofacial and dental regions [16]. One of the advancements AM has brought to implant manufacture is the design of predefined and controlled porosity to lower implant stiffness and reduce bone stress shielding [16, 17]. Porosity also enables bone ingrowth, further enhancing load transfer through the bone. The capability of bone ingrowth into porous structures has previously been shown in implants with porous coating, and various animal studies on additively manufactured porous titanium structures [10, 18]. The materials can be randomly or stochastically porous (e.g., foams), as well as controlled porous (e.g., lattice structures) to match the elastic modulus of the bone, which improves their long-term performance [12, 17, 19, 20].

Previous research has documented porous joint replacement designs, focusing especially on the design of porous materials and their performance [20–22]. However, to date, no review paper has focused on stress shielding in their comparison of different approaches for designing porous implants. This systematic review aims to provide an overview of design strategies used in additively manufactured porous orthopedic joint replacement implants to reduce bone stress shielding.

The evaluation of the designs in each of the studies regarding stress shielding and the consideration of the reduced mechanical strength were analyzed. This review of the current state of the art identifies existing design approaches and suggests further directions to address the issue of stress shielding caused by orthopedic implants.

## Materials and methods

### Search strategy

**Fig. 1:**
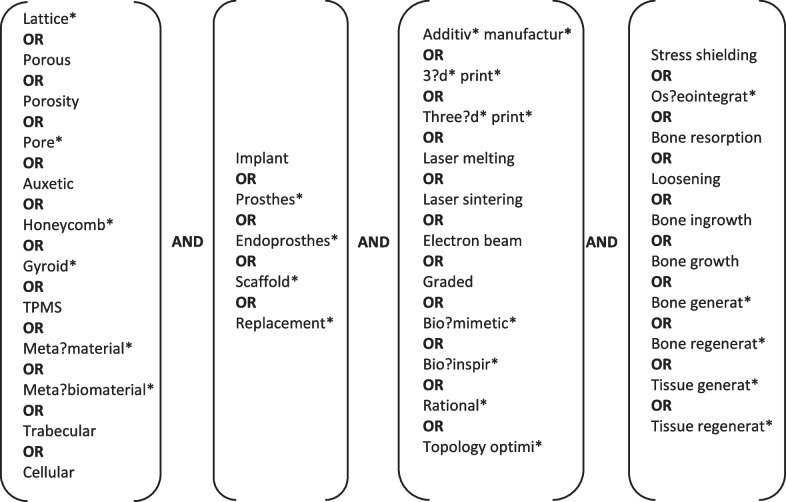
Keywords used in the search on Scopus, Web of Science, Medline, Embase, Compendex, and Inspec. The search string was of the form ‘(Lattice* OR Porous OR … OR Cellular) AND (Implant OR Prosthes* OR… OR Replacement*) AND (Additiv* manufactur* OR 3?d* print*OR … OR Topology optimi*) AND (Stress shielding OR Os?eointegrat* OR … OR Tissue regenerat*)’

A broad literature search was conducted to identify articles in which stress shielding in orthopedic joint implants and additively manufactured porous materials were addressed (initial search: 07 June 2021, additional search for new publications: 19 September 2022). This search was based on the following relevant keywords which were combined through AND operators: ‘lattice,’ ‘implant,’ ‘additive manufacturing,’ and ‘stress shielding.’ To ensure maximal coverage of relevant literature, synonyms and similar words were added to the search for each keyword and connected through OR operators (Fig. 1). Additionally, words describing the design approach or criteria, such as biomimetic and topology optimization, were added to the ‘additive manufacturing’ category to broaden the search to include studies that were solely computational. Masking and truncations were used to cover possible variations of the words. Due to the interdisciplinary nature of the research question, the search for relevant literature was conducted in six databases, namely Scopus, Web of Science, Medline, Embase, Compendex, and Inspec.

### Selection criteria

Duplicates were removed, and the remaining articles were screened for eligibility by two independent reviewers (S.S. and Y.Y.). Covidence systematic review software (Veritas Health Innovation, Melbourne, Australia) was used for the screening process. Firstly, titles and abstracts were assessed, and then, full texts of the selected articles were retrieved to determine whether they fulfilled the inclusion criteria. To be included in this review, the publications were required to (i) describe the design or the validation of a uncemented orthopedic joint replacement implant, (ii) use a controlled porous material, (iii) be additively manufactured, (iv) use metallic materials, (v) address and assess stress shielding, (vi) be peer-reviewed journal articles, and (vii) be written in English. In addition, we excluded (i) studies involving biodegradable scaffold materials, (ii) studies focusing on the influence of material composition, or heat or chemical treatment, and (iii) studies focusing on the influence of surface coating and modification. Review articles and meta-analyses were excluded. Articles describing the design and/or validation of the same implant over multiple publications, presented by the same research group, were combined and evaluated jointly as one study.

### Quality assessment

The quality of each of the included studies was assessed by one reviewer (S.S.) within the context of the current review using a modified questionnaire based on the Downs and Black checklist [23] and the STROBE statement [24]. Eleven questions were identified to determine the overall quality of the article for this review (Table 1). Each study was assigned a score of 2 (fully addressed), 1 (partially addressed), or 0 (not addressed) for each of the eleven questions. A total score for each article was calculated by summing up the relevant scores for the individual questions. Based on their total score, articles were classified as high quality (total score ≥ 20), moderate quality (20 > total score ≥ 15), or low quality (total score < 15).

**Table 1:** Questions for quality assessment based on the Downs and Black checklist [23] and the STROBE statement [24]

Number	Quality assessment question (QAQ)
1	Is the scientific background/rationale for the investigation reported?
2	Is the aim/objective of the study clearly described?
3	Is the porosity design method clearly described?
4	Is the rationale of the porosity design clearly described?
5	Is the study design clearly described?
6	Is the study design suitable to validate the porosity design with regard to stress shielding?
7	Are the outcome measures suitable to validate the porosity design with regard to stress shielding?
8	Are the outcome measures reliable?
9	Are the outcomes and main findings of the study clearly described?
10	Are the key results clearly stated regarding the study objectives?
11	Are the limitations of the study discussed?

### Data extraction

After conducting the search and assessing the articles for eligibility and quality, relevant data were extracted from all studies by one reviewer (S.S.). This included the type of implant, the design strategy to reduce stress shielding, and the lattice geometry used for the design. Additionally, outcomes as a result of porous design to mitigate stress shielding reduction were also documented. Inclusion of mechanical strength into the design and/or the validation was examined for each article.

## Results

### Search results and quality

**Fig. 2:**
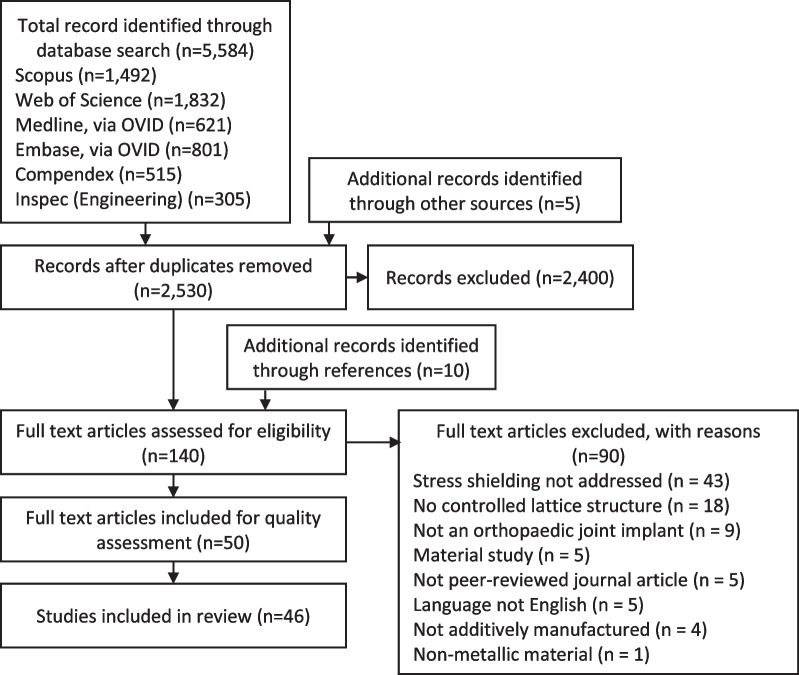
Flowchart of study selection from identified records

The total number of records identified through the extensive database search was 5584. Five additional papers that were identified during screening were also included. After removal of duplicates, 2530 titles, keywords, and abstracts were screened for eligibility. During this step, 2400 articles were excluded as they did not match the inclusion criteria. With an additional ten articles identified through reference search of included articles, the total number of articles for full-text assessment was 140. Applying the inclusion criteria during full-text screening, 90 articles were excluded because: they did not address stress shielding (*n* = 43), stochastic or random-based material was used rather than controlled porosity (*n* = 18), the implant was not an orthopedic joint replacement (*n* = 9), the study was focused on investigating material behavior (*n* = 5), the article was not peer-reviewed (*n* = 5), the article was not written in English (*n* = 5), the implant was not additively manufactured (*n* = 4), or the implant was non-metallic (*n* = 1). In total, 50 articles based on 46 studies were included in this systematic review (Fig. 2).

**Fig. 3:**
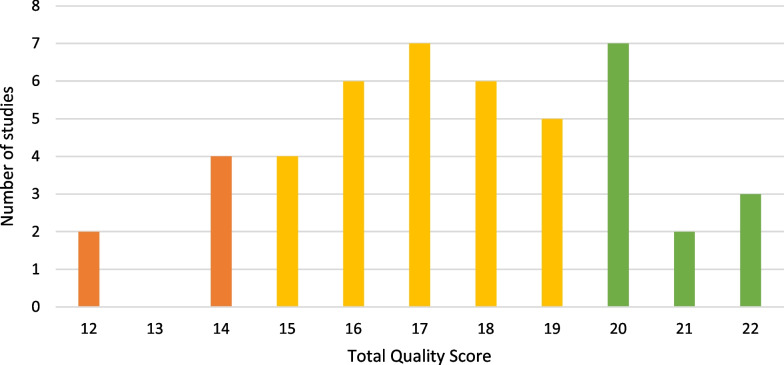
Number of studies over total quality score reached. Studies were of high (green), moderate (yellow), or low (orange) quality

Out of the 46 studies analyzed, most (61%, *n* = 28) were assessed to be of moderate quality, 26% (*n* = 12) were assessed to be of high quality, and 13% (*n* = 6) were assessed to be of low quality based on the thresholds given in the Methods section (Fig. 3). The mean total score over all studies was 17.5.

**Fig. 4:**
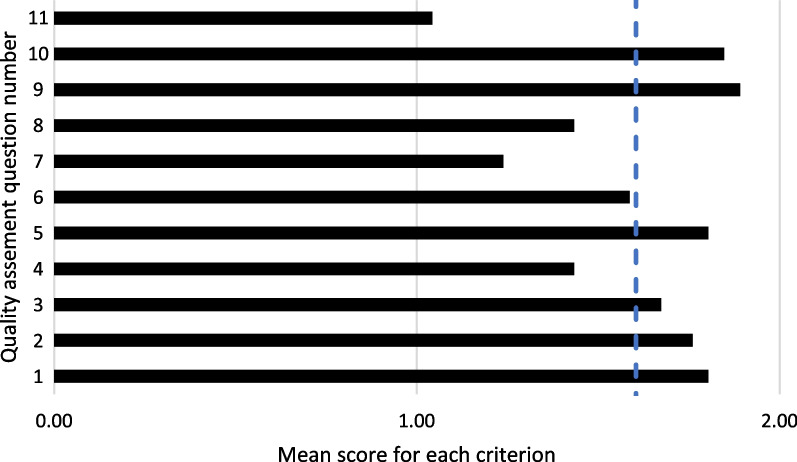
Mean score for each of the eleven quality assessment question (Table 1) across all studies included in the current review. The vertical dashed line indicates the overall mean score computed across all criteria

The overall mean score computed across all questions was 1.6 (Fig. 4). For four of the eleven questions, the mean score over all studies was below the mean, including the description of the rationale of the porosity and implant design (1.4), the suitability (1.2), and reliability (1.4) of the outcome measures used to evaluate stress shielding, and the discussion of the study’s limitations (1.0) (Fig. 4).

### Implant types

Most studies included in this review introduced porosity designs for hip and knee joint replacement implants. Only one of the included studies presented a porous design of a shoulder replacement. In 83% (*n* = 38) of the studies, components of a total hip replacement were presented, while 15% (*n* = 7) designed components of a total knee replacement. The hip femoral stem was the most reported on with 33 (72%) studies focusing on its design. Five (11%) studies focused on the acetabular cup design, two of which focused on cages for reinforcing the acetabular cup. Four studies (9%) focused on lattice designs for the tibial component of a knee replacement, one of which presented a design for a ‘block augmentation’ (an additional component used with a TKR in patients with a tibial bone defect). Two studies (4%) focused on designs of the femoral knee replacement component, one of which focused solely on the design of the pegs in the femoral component.

### Additive manufacturing

The design of porous structures with conventional manufacturing methods requires a different approach compared to the use of AM which allows for higher complexity in the internal structures. Therefore, only studies in which AM was considered suitable for the manufacturing of the presented implant design were included in this review. The most selected material was titanium in 87% of the studies (*n* = 40), followed by cobalt–chromium (7%, *n* = 3), functionally graded material combinations (7%, *n* = 3), and stainless steel (4%, *n* = 2) (Table 2). Almost half of the included studies (46%, *n* = 21) did not report a specific AM technology for their porous design. SLM was used or intended to be used for manufacturing in 35% of the studies (*n* = 16), followed by EBM (9%, *n* = 4), Direct Metal Laser Sintering (DMLS, 9%, *n* = 4), and Direct Metal Printing (DMP, 2%, *n* = 1).

**Table 2:** Materials and AM technologies selected for the porous implant design

Study	Material	Selected AM technology
[30]	Titanium (alloy)	SLM
[25]	Titanium (alloy)	SLM
[56]	Titanium (alloy)	SLM
[58]	Titanium (alloy)	SLM
[69]	Titanium (alloy)	SLM
[51]	Titanium (alloy)	SLM
[59]	Titanium (alloy)	SLM
[60]	Titanium (alloy)	SLM
[52, 53]	Titanium (alloy)	SLM
[61]	Titanium (alloy)	SLM
[34]	Titanium (alloy)	SLM
[38]	Titanium (alloy)	SLM
[41]	Titanium (alloy)	SLM
[63]	Titanium (alloy)	SLM
[99]	Cobalt-chrome	SLM
[15]	Cobalt-chrome	SLM
[57]	Titanium (alloy)	EBM
[81]	Titanium (alloy)	EBM
[40]	Titanium (alloy)	EBM
[36, 37]	Titanium (alloy)	EBM
[31]	Titanium (alloy)	DMP
[26, 42]	Titanium (alloy)	DMLS
[14, 27]	Titanium (alloy)	DMLS
[33]	Stainless steel	DMLS
[29]	Cobalt-chrome	DMLS
[48]	Titanium (alloy), stainless steel, functionally graded material (FGM)—titanium, stainless steel	–
[84]	Titanium (alloy)	–
[43]	Titanium (alloy)	–
[47]	Titanium (alloy)	–
[39]	Titanium (alloy)	–
[44]	Titanium (alloy)	–
[50]	Titanium (alloy)	–
[64]	Titanium (alloy)	–
[49]	Titanium (alloy)	–
[67]	Titanium (alloy)	–
[46]	Titanium (alloy)	–
[55]	Titanium (alloy)	–
[28]	Titanium (alloy)	–
[65]	Titanium (alloy)	–
[62]	Titanium (alloy)	–
[45]	Titanium (alloy)	–
[66]	Titanium (alloy)	–
[35]	Titanium (alloy)	–
[68]	Titanium (alloy)	–
[54]	Functionally graded material (FGM)—titanium, alumina ceramic	–
[70]	Functionally graded material (FGM)—titanium, alumina ceramic	–

### Implant design testing

**Fig. 5:**
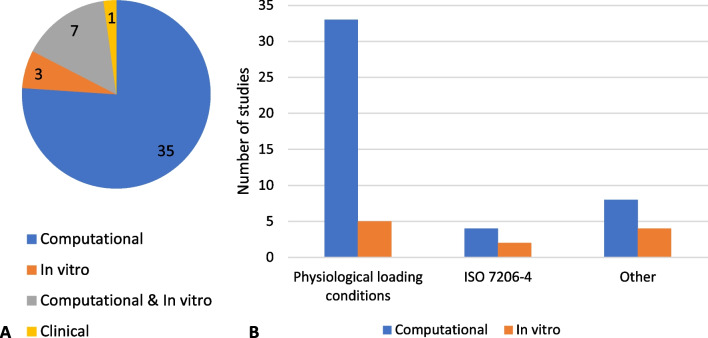
Approaches to evaluate stress shielding and mechanical properties: **A** Number of studies conducted computationally, in vitro, or clinically, and **B** number of studies using setups with physiological loading conditions, models using an ISO 7206–4 setup, and other setups in computational and in vitro models

All studies included in this review conducted experiments or analyses to evaluate their designs in relation to stress shielding and mechanical performance (Tables 3, 4 and 5). One of the studies was clinically based in which radiographic data from a hospital database were evaluated, while the remaining 45 studies examined the presented designs through finite element analysis or in vitro experiments. Most studies included (76%, *n* = 35) were solely computational studies, followed by studies conducting computational and in vitro experiments (15%, *n* = 7) and solely in vitro experiments (7%, *n* = 3) (Fig. 5A). Many of the included studies (72%, *n* = 33) used physiological loading in their models and experimental protocols. Other studies (9%, *n* = 4) used the loading conditions described in the ISO 7206–4 standard for hip implants (Fig. 5B).

**Table 3:** Implant type, design and validation strategies, and stress shielding measured of studies applying uniform porosity designs

Study	Implant type	Lattice design	Main design parameter	Computational validation	Experimental validation	Main stress shielding measure	Max. stress shielding reduction ^*^	Overall quality score
[81]	Hip acetabular cup	Extensive porous surface	Pore size, porosity, elastic modulus	–	Clinical study, radiographic assessment	Stress shielding grading system	Absent − 60% Mild + 58% Moderate − 100% Severe + 100%	17
[33]	Hip femoral stem	Simple gyroid—solid outer skin	Elastic modulus	–	–	Elastic modulus	Not quantified	14
[39]	Hip femoral stem	Auxetic—porous proximal part	Elastic modulus	Implanted model	–	Stress and strain in bone	Stress + 27% Strain + 83%	18
[40]	Hip femoral stem	Cubic—porous proximal part	Pore size	Implanted model	–	Stress and strain in bone	Stress + 7% Strain + 15%	17
[36, 37]	Hip femoral stem	Rhombic dodecahedron—fully porous	Elastic modulus	Implanted model	Flexure testing, implanted model	Stress in bone, bone surface strains	Not quantified	18
[29]	Hip femoral stem	Cubic—solid outer skin	Strut size	Bending test	Bending test	Bending stiffness	Bending stiffness − 60%	16
[26, 42]	Hip femoral stem	Diamond—solid outer skin except for proximal part	Elastic modulus	ISO 7206-4, implanted model	Implanted model	Bone resorption	Formation − 18% Homeostasis + 6% Resorption -19%	22
[14, 27]	Hip femoral stem	Body-centered cubic (BCC)—solid outer skin with beads in proximal part	Porosity	ISO 7206-4, implanted model	–	Stress in bone, stress shielding increase	Gruen zone 7: − 28% SSI − 90%	19
[35]	Hip femoral stem	Body-centered cubic (BCC)	Mechanical properties	Implanted model	–	Stress shielding signal (SSS)	SSS − 81% (up to)	18
[41]	Hip femoral stem	Face and body-centered cubic with z-truss (FBBCz), Octet-truss	Elastic modulus, yield strength	Structural analysis	–	mechanical properties	Not quantified	14
[34]	Hip femoral stem	Body-centered cubic (BCC)	Density	Implanted model	–	Stress shielding signal	Gruen zone 6: − 11%, Gruen zone 7: − 25%	19
[43]	Knee femoral component pegs	–	Porosity of geometrically optimized pegs	Implanted model	–	Stress in bone	Stress + 18.16%	16
[44]	Knee tibial component	Rhombic dodecahedron—fully porous stem	Pore size, porosity	Implanted model	–	Stress and strain energy in bone	Stress + 64% Strain energy + 121%	12
[45]	Knee tibial component	Gyroid	Elastic modulus	Implanted model	–	Stress and strain energy in bone	Stress + 30%, strain energy + 91%	14
[38]	Shoulder humeral stem	Face-centered cubic (FCC)	Mass reduction	–	–	Elastic modulus	Not quantified	14

**Table 4:** Implant type, design and validation strategies, and stress shielding measured of studies applying graded porosity designs

Study	Implant type	Porosity type	Main design strategy	Computational validation	Experimental validation	Main stress shielding measure	Max. stress shielding reduction	Overall quality score
[31]	Hip acetabular cup	Diamond, body- centered cubic, rhombic dodecahedron	3 porosity-graded layers from inside to bone–implant interface	–	Compression testing, DIC	Strains, deformation pattern	Not quantified	17
[84]	Hip femoral stem	Body‐centered cubic (BCC)	9 axially porosity-graded layers	Implanted model	–	Stress in bone	Gruen zone 7: + 50%	19
[47]	Hip femoral stem	Schwarz Primitive	9 axially porosity-graded layers	Implanted model	–	Stress in bone	Not quantified	16
[50]	Hip femoral stem	Auxetic	Positive to negative Poisson’s ratio from one side to another	Implanted model	–	Stress and strain in implant	Not quantified	20
[99]	Hip femoral stem	Square	2 axially porosity-graded layers	Implanted model	–	Stress in bone	Gruen zone 2: + 22% Gruen zone 3: + 18% Gruen zone 5: + 20% Gruen zone 6: + 36% Gruen zone 7: + 12%	20
[48]	Hip femoral stem	–	Porosity gradually decreased toward distal end	Implanted model	–	Stress in bone	*Cortical*: Steel: 29%, Titanium: + 21%, FGM: + 21% *Cancellous*: Steel: + 14%, Titanium: + 10%, FGM: + 15%*^1^	15
[51]	Hip femoral stem	Auxetic, honeycomb	Positive to negative Poisson’s ratio from one side to another	–	Simplified implanted model	Strains in bone	Not quantified	15
[15]	Hip femoral stem	Octahedral	5 axially porosity-graded layers, 3 radially porosity-graded layers	Implanted model	Bending test, implanted model	Stress in bone	Gruen zone 7: + 368%	16
[49]	Hip femoral stem	–	Axially, radially and combined porosity-graded	Structural analysis	–	Stress shielding effect	SSE -31%	15
[52, 53]	Hip femoral stem	Variations of body-centered cubic (BCC)	Strut thickness based on interaction with cortical/trabecular bone, alternating pore size	Implanted model	–	Stress in implant	Not quantified	16
[46]	Hip femoral stem	Diamond	3 radially porosity-graded layers, 4 axially porosity-graded layers	Implanted model	–	Bone loss	Bone loss − 75%	20
[28]	Hip femoral stem	Cubic	3 radially porosity-graded layers	ISO 7206-4, implanted model	–	Stress in bone	Gruen zone 7: + 65%	17
[54]	Knee femoral component	–	Porosity decrease from uppermost to lowermost surface	Implanted model	–	Stress in bone (interface)	Stress + 41.5%	18
[55]	Metal block augmentation for knee tibial component	Cubic/grid	2 porosity sections based on topology optimization	Implanted model	–	Stress in bone	Stress + 18.60%	19

*^1^The materials in this study were all porosity-graded, and FGM is referring to the combination of two materials (steel and titanium). The stress shielding measure chosen is the stress increase in the surrounding bone and reported separately for the cortical and cancellous area

**Table 5:** Implant type, design and validation strategies, and stress shielding measured of studies applying optimized porosity designs

Study	Implant type	Porosity type	Optimization method	Computational validation	Experimental validation	Main stress shielding measure	Max. stress shielding reduction*	Overall quality score
[30]	Hip acetabular cup	Vintiles	Minimize material, sufficient mechanical strength	Model including femoral head	–	Weight reduction	Weight − 69%	12
[59]	Hip cage for acetabular reinforcement	Tetrahedron	Minimize compliance, relative density, volume fraction to enable bone ingrowth	Implanted model	–	Stress, strain energy in implant	Stress − 43% Strain energy − 3.88% Strain energy − 3.88%	20
[62]	Hip cage for acetabular reinforcement	Octet-truss	Minimize compliance	Implanted model	–	Stress in implant	− 75%	17
[25]	Hip femoral stem	Vintiles	Minimize material, sufficient mechanical strength, porosity, stress	ISO7206-4	ISO7206-4	Weight reduction	Weight − 50%	15
[56]	Hip femoral stem	Square	Minimize bone loss, interface failure, porosity	Implanted model	–	Bone loss	Bone loss − 76%	21
[57]	Hip femoral stem	Square, Kagome	Minimize bone loss, interface failure, fatigue safety factor, porosity	Implanted model	–	Bone loss	Bone loss − 58%	22
[58]	Hip femoral stem	Tetrahedron	Minimize bone resorption, interface failure, safety factor	Implanted model	Implanted model	Volumetric and surface bone loss	Bone loss − 75%	22
[69]	Hip femoral stem	Elastically isotropic, cubic cross, smoothed cubic cross	Minimize shear stress with bone resorption < 0.05	Simplified implanted model	–	Shear stress	Not quantified	18
[64]	Hip femoral stem	–	Minimize SSI, yield stress, shear stress, elastic modulus	Implanted model	–	Stress shielding increase	SSI − 7–11%	18
[67]	Hip femoral stem	–	Optimized radially graded: increase elastic modulus of elements until minimum element safety factor > global safety factor	Implanted model	–	Bone loss	Bone loss − 40%	20
[61]	Hip femoral stem	Tetrahedron	Maximize compliance, fatigue safety factor, interface failure, relative density	Implanted model	–	Bone loss	Bone loss − 58.1%	20
[66]	Hip femoral stem	Modified body-centered cubic (BCC)	Maximize stress in femur in 4 GZs to find elastic modulus in 8 segments	Implanted model	Implanted model	Stress in bone	Gruen zone 7: + 42.8%	16
[63]	Hip femoral stem	Modified cubic (negative, neutral, positive Poisson's)	Minimize bone resorption and interface stress	Implanted model	–	Bone loss	− 64%	21
[65]	Hip femoral stem	Body-centered cubic (BCC)	Minimize stress shielding and compliance	Implanted model	–	Stress in bone	+ 32.40%	17
[68]	Hip femoral stem	Body-centered cubic (BCC)	Minimize bone loss and iterations	Implanted model	–	Bone loss	Not quantified	20
[70]	Knee femoral component	–	Weighted optimization of FGM: composition and porosity parameters, subject to stress in bone, micromotion, wear	Implanted model	–	Stress in bone	Stress + 3.8%	17
[60]	Knee tibial component	Tetrahedron	Minimize stiffness—maximize compliance, fatigue safety factor, density	Implanted model	–	Bone resorption	Bone resorption − 26%	19

*The stress shielding increase can refer to either an increase or decrease in the stress shielding measure chosen. This is indicated by + for increase or—for decrease

#### Stress shielding

**Fig. 6:**
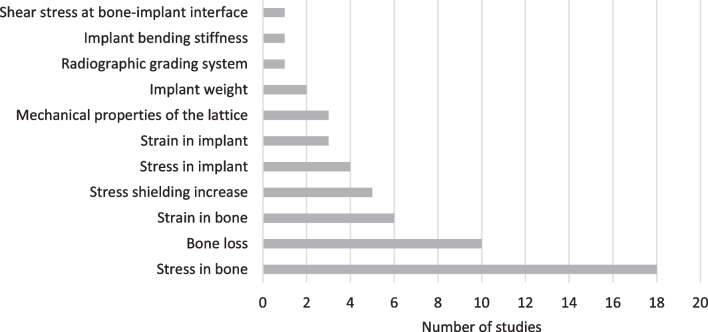
Main parameter used to evaluate the impact of the presented design on the stress shielding

In the included studies, different measures were used to quantify the effect of the presented design on stress shielding (Fig. 6). Most studies (39%, *n* = 18) used bone stress followed by bone loss (22%, *n* = 10) and bone strain (13%, *n* = 6). Mechanical properties (7%, *n* = 3) and implant weight (4%, *n* = 2) were also considered for the evaluation of stress shielding in some studies.

To quantify the reduction of stress shielding, stress and strain values were either interpreted directly or used for the calculation of other measures such as the stress shielding increase (SSI) (Tables 3, 4 and 5), comparing the relative change in stress (or strain) in the bone before and after implantation. Bone remodeling was found to be a recurring measure used to evaluate stress shielding among the studies. Strain energy density in bone before and after implantation is compared to determine the regions of bone loss, growth, and homeostasis [13].

#### Mechanical strength

For the validation of the performance of hip implant designs, a setup following the ISO 7204-6 standard was used in four studies [25–28]. Other studies selected physiological or non-physiological loading conditions in their computational models or experiments. Non-physiological experiments for the validation of the mechanical strength included flexure/bending tests on femoral hip stems [15, 29] and compression testing of the acetabular cup [30, 31]. The mechanical strength of the material was either compared to values observed in the mechanical analysis of the implant (53%) or included in the optimization scheme (31%). However, seven (16%) studies did not consider mechanical strength as a design parameter. Only nine (20%) studies considered fatigue in the design and/or validation of their lattice structure.

Porous structures were often simplified using representative volume elements (RVE) and modeled as continuums in finite element models rather than modeling their detailed porous geometry [32]. RVEs were used in 33 (73%) of the selected studies (Table 6).

**Table 6:** Mechanical strength validation

Study	Implant type	Computational analysis			Experimental analysis		Mechanical strength as design criteria			
		Structural analysis lattice	Structural analysis implant	Representative volume element (RVE)	Mechanical testing lattice	Mechanical testing implant	Included in optimization	Comparison to yield strength	Fatigue strength considered	Not addressed
[30]	Hip acetabular cup		✓				✓			
[81]	Hip acetabular cup									✓
[31]	Hip acetabular cup				✓	✓		✓		
[59]	Hip cage for acetabular reinforcement		✓	✓			✓			
[62]	Hip cage for acetabular reinforcement		✓	✓	✓		✓			
[25]	Hip femoral stem		✓			✓	✓			
[84]	Hip femoral stem	✓	✓	✓				✓		
[56]	Hip femoral stem		✓	✓			✓			
[57]	Hip femoral stem		✓	✓			✓		✓	
[58]	Hip femoral stem		✓	✓			✓		✓	
[47]	Hip femoral stem	✓	✓	✓						✓
[33]	Hip femoral stem				✓			✓		
[69]	Hip femoral stem		✓	✓						✓
[39]	Hip femoral stem	✓	✓	✓				✓		
[40]	Hip femoral stem		✓	✓	✓			✓		
[50]	Hip femoral stem		✓	✓				✓		
[36, 37]	Hip femoral stem		✓	✓	✓	✓		✓		
[29]	Hip femoral stem		✓	✓		✓		✓		
[99]	Hip femoral stem		✓	✓				✓		
[48]	Hip femoral stem		✓	✓						✓
[26, 42]	Hip femoral stem		✓	✓		✓		✓		
[51]	Hip femoral stem				✓	✓		✓		
[15]	Hip femoral stem		✓	✓		✓		✓		
[14, 27]	Hip femoral stem	✓	✓	✓				✓	✓	✓
[64]	Hip femoral stem		✓	✓			✓			
[49]	Hip femoral stem		✓	✓				✓		
[52, 53]	Hip femoral stem		✓					✓		
[67, 68]	Hip femoral stem		✓	✓			✓		✓	
[46]	Hip femoral stem		✓	✓				✓	✓	
[61]	Hip femoral stem		✓	✓			✓		✓	
[34]	Hip femoral stem			✓		✓				
[28]	Hip femoral stem	✓	✓	✓		✓				
[65]	Hip femoral stem		✓	✓	✓		✓			
[41]	Hip femoral stem			✓		✓				
[63]	Hip femoral stem		✓	✓	✓					
[66]	Hip femoral stem		✓	✓						
[35]	Hip femoral stem		✓	✓		✓				
[68]	Hip femoral stem		✓	✓			✓		✓	
[54]	Knee femoral component		✓	✓						✓
[70]	Knee femoral component		✓	✓						✓
[43]	Knee femoral component pegs		✓	✓				✓		
[44]	Knee tibial component		✓					✓		
[60]	Knee tibial component		✓	✓			✓		✓	
[45]	Knee tibial implant			✓		✓				
[55]	Metal block augmentation for knee tibial component		✓	✓	✓					
[38]	Shoulder humeral stem	✓		✓		✓				

### Porosity designs

**Fig. 7:**
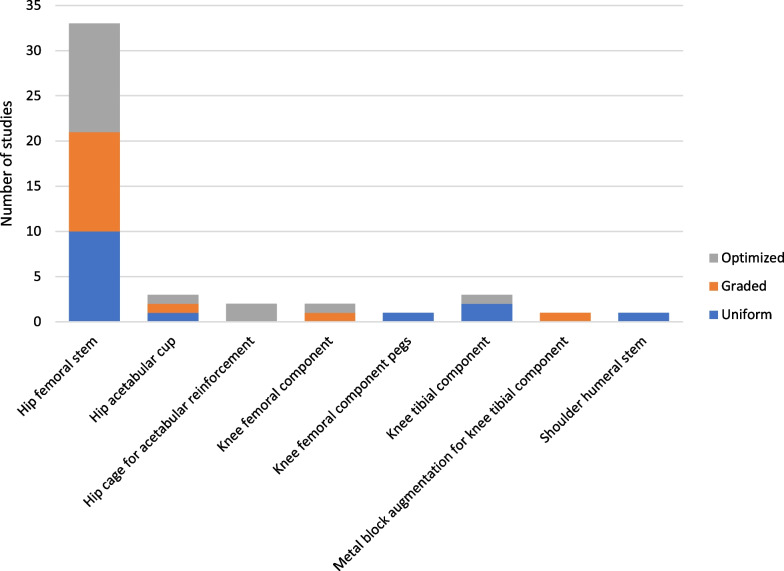
Number of studies conducted on each type of implant and porosity design strategies

Porous implants were broadly categorized into three groups based on the distribution of porosity within the implant. These groups were: uniform porosity, graded porosity, and optimized porosity (Fig. 7).

**Fig. 8:**
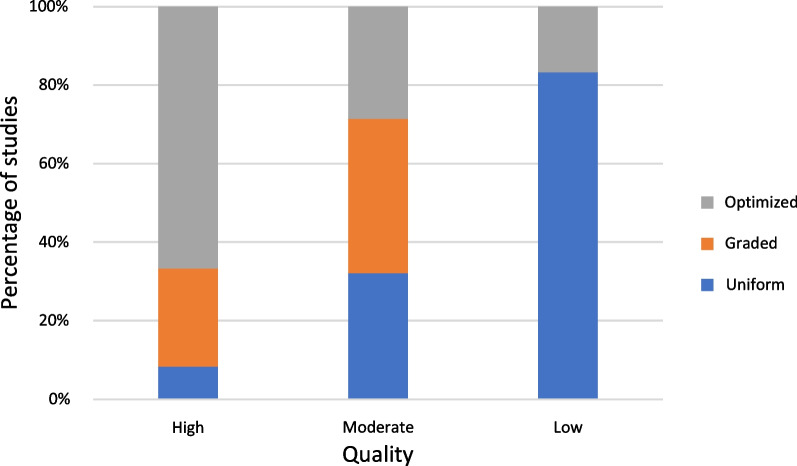
Quality distribution between porosity design groups in per cent

Studies using optimized porosity for their porous implant design exhibited the highest quality (mean quality score 18.5), followed by graded porosity (mean quality score 17.4) and uniform porosity (mean quality score 16.5). The amounts of studies of high, moderate, and low quality are highest for the optimized group (67%, *n* = 8), the graded group (39%, *n* = 11), and the uniform group (83%, *n* = 5), respectively (Fig. 8).

#### Uniform porosity

Uniform porosity, characterized by the use of a single unit cell design repeated throughout all porous areas of the implant, was used in 15 (33%) of the included studies (Table 3). The aim when using a uniform porosity design was to match typical, average mechanical properties of bone as closely as possible. A variety of unit cell designs were applied to achieve this aim.

While some of the presented implants had a solid shell surrounding the porous structure [29, 33, 34], most studies used open porosity to enable bone ingrowth. The extent of open porosity in the designed implants varied. In two studies, fully porous hip replacements were designed with only the neck of the femoral component being left solid [35–37]. Similarly, almost fully porous shoulder implants were investigated in another study [38].

Eldesouky et al. (2017) reported a reduction of stress shielding through the inclusion of a cubic lattice into the proximal part of the implant, which was further improved in a later study using auxetic lattice structures [39, 40]. Another design facilitating boney ingrowth in the proximal part of the stem was presented by Mehboob et al. (2020) who used beads to create an open porosity in their encapsulated body-centered cubic lattice design [14, 27]. Designs with partial open porosity in mostly the proximal part were investigated in another study to determine the most suitable design space to reduce stress shielding while not exceeding the material’s yield stress [41]. Jette et al. (2018) left an open window in the solid shell of their stem to facilitate osseointegration [26, 42]. Reduced stress shielding was also shown in knee replacements with uniform porosities in the stem of tibial components in two studies, and the geometrically optimized pegs of a femoral component in another study [43–45].

#### Functionally graded porosity

Defined gradients with a specified number of different unit cell designs that are distributed rationally based on assumptions obtained from analysis and the literature are summarized as functionally graded designs. Functionally graded porous materials were used in 14 of the included studies (Table 4). However, five of them do not present an actual lattice design but rather a graded stiffness. Since the chosen material properties were associated with porous/lattice materials, they can be directly translated into such designs. Moreover, 36% did not quantify the reduction in stress shielding explicitly but instead compared stress or strain distributions to qualitatively describe the improvement.

Comparisons between radial and axial grading of hip stems in two studies suggest that both approaches can be effective in reducing stress shielding [15, 46]. While the radial grading which implemented a stiffer outer shell was found to better mitigate stress shielding than a proximally stiffer axial grading, the observed differences were not significant [15, 46]. Similar to these results, other studies found that decreasing stiffness toward the distal end improved the load exposure of the bone, while a stiffness increase toward the distal end resulted in a better load bearing performance of the implant and improved stress distribution in bone [47]. In contrast, Hedia et al. (2019) compared the influence of porosity in their study but also material grading with a porosity decrease toward the distal end and reported a stress increase in the femur [48]. Al Zoubi et al. (2022) compared radially graded and uniform designs with different porosities and found that designs with increasing porosity toward the center of the implant performed best in terms of stress transfer to bone, but also micromotion and mechanical strength [28]. Singh et al. (2018) reported that a combination of both, radial and axial grading, could further enhance the decrease in stress shielding and the increase in bone ingrowth [49].

Besides axial and radial grading, other approaches for functionally graded porosity design in femoral stems were identified amongst the included studies. Two studies presented a hip stem design with lattice structures in the proximal area, combining design with negative and positive Poisson’s ratio to expose the femur bone to compression on both sides of the implant [50, 51]. Sufiiarov et al. (2021) changed the parameters of their lattice structure depending on which parts of the implant interacted with cortical and trabecular bone and reported a favorable stress distribution in the femur compared to a solid implant [52, 53].

In addition, functionally graded porosity was found to be beneficial in other implant components. Kolken et al. (2021) studied various lattice structures and applied them for the design of porous acetabular cups with higher porosity at the bone–implant interface to fully dense material at the joint revealed a superior performance of the diamond lattice structure [31]. In one study, a stress increase at the bone–implant interface was achieved using a graded porous knee implant design without a specific lattice design by gradually changing the porosity from one surface to the other [54]. Another study proposed a porous design for metal block augmentation for the tibial component of a knee replacement to address stress shielding [55]. Design spaces for higher and lower porosity were selected through topology optimization with the aim to achieve sufficient mechanical strength despite material reduction [55].

#### Optimized porosity

Optimized porosity refers to the use of optimization algorithms to achieve the most suitable design specification. Optimization to reduce stress shielding was employed in 17 of the included studies (Table 5). While not all the studies used a customized approach in their optimization, 65% (*n* = 11) used the mechanical properties of bone obtained from CT scans to optimize the implant internal structure.

A recurring objective in optimized porosity design approaches was the reduction of bone loss to target the effects of stress shielding directly. Arabnejad et al. (2012, 2013, 2017) minimized bone loss subject to interface failure, a defined safety factor and porosity in a hip stem [56–58]. Based on their findings, optimizations on a hip stem, a hip cage, and a knee implant were conducted using material compliance as the main design factor increasing the amount of stress and strain exposure in the bone and reducing bone resorption [59–61]. Similarly, Xu et al. (2022) minimized compliance for the design of their Burch–Schneider cage [62]. Minimizing bone resorption but also interface stress, Garner et al. (2022) presented a porous femoral component with varying Poisson’s ratio [63]. This study was the only one included in this review using optimization to design an implant with auxetic lattice structures.

Other than bone resorption, bone stress was targeted in the optimization in some studies. Saravana et al. (2017) used the comparison of stress present in the femur before and after implantation, namely SSI, as their objective function taking yield and stress, and elastic modulus into consideration for the optimization [64]. A similar approach was applied in a study presenting a hip stem design based on optimized stress shielding and compliance [65]. Gao et al. (2022) designed a femoral stem consisting of eight porous segments maximizing the stress transferred to the surrounding bone [66]. In contrast to other studies, Sun et al. (2018) started the optimization process from a fully porous, low-stiffness implant and iteratively increased the stiffness of each mesh element until the global safety was reached [67]. In a later study, Sun et al. (2022) considered the nonlinear relationship between the elastic modulus and the stress distribution and minimized bone loss to determine a suitable lattice design for their femoral component [68].

To target implant failure, Cramer et al. (2017) focused on minimizing shear stress in their study and considered bone loss as a constraint [69]. Abate et al. (2019, 2021) targeted material reduction considering sufficient mechanical strength, porosity, and strut diameter reducing the weight significantly compared to a solid implant [25, 30]. In another study, Bahraminasab et al. (2014) based their optimization on the findings from their previous study and developed a weighted optimization method for a femoral knee component [70]. Through the graded materials considering composition and porosity parameters subject to stresses in the surrounding bone, micromotion, and implant wear, the stress in the bone increased [70].

## Summary and discussion

The emergence of additive manufacturing in the medical device industry has a new capability to design joint replacement components with properties that match that of the underlying bone. Using lattice designs, porosity may provide a much larger range of mechanical properties that may reduce implant failure due to effects such as stress shielding. This systematic review aimed to identify design strategies presented in the literature and to suggest further directions to reduce stress shielding in joint replacement implants.

There were 46 studies identified that fit the inclusion criteria, most of which (*n* = 28) were of moderate quality. While the background and rationale of the investigations were mostly well described, many studies (*n* = 25) did not or only partially explain the rationale of the implant and lattice design strategy. Therefore, the reason for choosing a particular porous design and porosity distribution remained unclear. While the study design was well described in nearly all articles (*n* = 37), the reported outcome measures relating to stress shielding such as stress and/or strain in the surrounding bone were not fully analyzed and quantified (61%). For instance, a comparison of stress or strain maxima or minima instead of their distribution throughout the bone was found to be insufficient to determine the reduction of stress shielding. Furthermore, more than half of the studies (54%) did not report all their results nor compare them to outcomes reported in the literature. However, in most studies, the main findings were summarized (89%), and key results stated with regard to the study objective (85%). The lowest quality scores were achieved in the discussion of limitations. Several articles were found to not sufficiently discuss the limitations of their study (61%).

Apart from one clinically based study, all included studies evaluated their implant designs using computational and/or physical in vitro models. Computational models offer a cost-effective approach for trialing numerous designs and loading conditions; however, to provide physiologically valid predictions, model outputs must agree with experimental data. Seven studies followed this approach where model predictions were compared to physical tests of the matching implant under identical loading conditions [15, 25, 29, 37, 42, 58, 66]. For the remaining 35 studies, model outputs were not compared to in vitro models, which raises questions regarding the validity of the models. While computational models can allow for efficient testing of new implant designs, there is a need for experimental validation to evaluate their accuracy.

Another aspect regarding the validation of new implant designs is the quantification of stress shielding. While most studies provided stress and strain distribution within the bone and/or the implant under loading (*n* = 38), not all studies quantified the reduction of stress shielding. The location and magnitude of stress and/or strain maxima in the bone and/or implant may be a good indicator for the success of the new design. However, quantitative data comparing stress and strain in the bone and/or implant before and after implantation can provide a better understanding of the reduction of stress shielding and therefore bone loss related to a new implant design. Particularly, the inclusion of bone modeling and remodeling measures may accommodate the prediction of clinical outcomes with regard to stress shielding.

A key challenge with both computational and in vitro techniques is modeling all the physiological conditions experienced by the implant. Both approaches implemented loads that were representative of those in vivo, specifically loads obtained from instrumented hip and knee implants [71–79]. However, in all but one of the reviewed studies biological effects such as the composition of synovial fluid were not applied, yet these conditions are known to affect implant performance [80].

The study by Castagnini et al. (2019) [81] was a clinical based study where 3D printed porous implants were implanted in patients, thus exposing them to biological effects in vivo. Comparing a conventionally to an additively manufactured porous acetabular cup design, the authors found no significant differences in the midterm outcomes with regard to stress shielding and signs of osseointegration [81]. Clinical studies that did not assess stress shielding were excluded from this review. It is worth noting that the reproducibility of determining the extent of stress shielding based on radiographic bone loss assessment has been found to be limited as results varied between the assessing surgeons [82]. Even though more in vivo studies are needed to overcome the limitations of computational and in vitro modeling, the clinical evaluation of stress shielding remains a challenge in clinical settings.

For the included studies in this review, implant porosity was implemented using either a uniform (33%), graded (30%), or optimized (37%) design strategy. All studies reported stress shielding reduction based on the chosen measure of stress shielding. It was found that quality assessment of included studies showed 67% of high-quality studies used an optimized design strategy compared to 25% for graded and 8% for uniform porosity designs. This suggests higher reliability of the findings of studies on optimized porosity design approaches regarding reducing stress shielding. However, since the studies did not quantify the reduction of stress shielding in an identical manner, the superiority between the lattice design strategies remains unclear.

Although stress shielding was the focus of this review, there are other factors that influence implant longevity. For example, micromotion between implant and bone is known to impact bony ingrowth and, if excessive, can lead to implant loosening [83]. Reducing the stiffness mismatch to achieve a reduction of micromotion was a rationale for employing different design approaches [50, 59, 60]. Implant designs with uniformly porous materials were found to lead to higher micromotion at the bone–implant interface, negatively impacting the osseointegration [46, 67, 84]. Using a mechano-regulation algorithm to simulate bone tissue growth, Tarlochan et al. (2018) found porous functionally graded material to be superior to homogenously porous materials in terms of bone ingrowth and therefore fixation of the implant [85]. This finding indicates that graded porosity designs have a greater capability to enhance implant longevity than uniform designs.

Mechanical strength is an important factor in the longevity of an implant, since insufficient strength may lead to implant failure and, ultimately, revision surgery. Fatigue performance, which influences implant longevity, was factored into topology optimization schemes in several studies. This included a fatigue safety factor as a boundary condition [57, 58, 60, 61], optimizing the design until a suitable factor of safety was achieved [67], or by comparing the occurring stress in the porous design to the respective fatigue strength of the selected material [62, 65]. Considering fatigue strength in the optimization as a boundary condition enables the design of a graded porous design with reduced stiffness and sufficient mechanical strength.

Manufacturing imperfections impact mechanical performance and can cause premature material failure, which can be especially prevalent for porous structures produced by additive manufacturing [86]. Yet none of the included studies considered these defects. A compensation strategy may help reduce the geometrical and mechanical differences after manufacturing compared to the computational model [87]. Furthermore, Moussa et al. (2021) presented an approach to factor such compensation strategies into a topology optimization to achieve less disparity between computational designs and manufactured components [88]. These studies highlight the importance of considering the discrepancy in the geometry between designed and manufactured lattice because it results in significant discrepancies in mechanical properties between the designed and manufactured lattice.

Besides considering mechanical performance in porous implant designs, mechanical strength can be enhanced to avoid implant failure. Strategies to improve the mechanical performance, in particular fatigue strength, of additively manufactured lattice structures such as post-manufacturing treatments [89, 90], design adjustments (e.g., filleted nodes) [91], or manufacturing parameters (e.g., layer thickness, laser/electron beam power) of the lattice have been presented in the literature [92]. None of the studies included in this review considered these post-processing steps in their implant design. To prevent material failure in porous implants, strategies to enhance mechanical performance need to be understood and considered in the design.

The mechanical properties and surface finishing of porous implants impact remodeling of bone after implantation, which impacts their long-term performance. Enabling bone tissue growth into the implant for better fixation is a common rationale for introducing porosity [19]. An analysis of bone formation after implantation, however, was only found in the computational study by Mehboob et al. who analyzed the influence of implant stiffness on bone formation [14]. To enhance bone ingrowth, surface coatings or treatments ought to be considered for additively manufactured porous implants since they have been found to be beneficial [18, 93, 94]. Looking at time-dependent and time-independent algorithms, Wu et al. found that the formation of bone tissue and the resulting change of load transfer had a significant impact on the design of their topology optimized scaffolds [95]. This finding indicates the importance of considering time-dependent mechanobiological models in implant design for osseointegration [95].

This review had limitations that ought to be acknowledged. Notably, studies on cemented implants were excluded in this review. Cementless implants are used to preserve the underlying bone for cases where revisions are more likely, such as in younger patients who have a longer life expectancy and are therefore more likely to outlive their implant [4]. Since cementless implants require greater bone stock for fixation, avoiding bone resorption is of greater concern for these types of implants compared to cemented. While it is feasible that implant porosity may be designed to enhance cemented implant performance, this would require different design criteria involving cement–implant interaction.

Studies on porous structures for other types of implants than joint replacements were excluded from this review. Approaches using lattice structures to reduce stress shielding can also be found in other types of bone replacement implants and dental applications [16, 96–98]. However, due to the multi-axial, dynamic, and high loading conditions at joints, the requirements of joint replacements differ from other bone-interfacing implants such as those for the treatment of large bone defects. Additionally, other factors besides stress shielding affecting implant longevity, including wear [54, 70] and micromotion [14, 15, 28, 35, 44, 46, 50, 59–61, 63, 70, 84], were not considered in the analyses.

## Conclusion

The longevity of joint replacement implants is adversely affected by a stiffness mismatch between implant and the surrounding bone. Introducing porosity into stiff implant materials, facilitated by additive manufacturing technologies, has become an area of interest to address the issue of stress shielding and enable osseointegration. Functionally grading material properties through lattice structures, especially through optimization, can be used to reduce the effects of stress shielding under consideration of the resulting increase in micromotion and decrease in mechanical strength. Due to a lack of consistent validation and quantification of stress shielding, no superior porosity design strategy has been identified to date. Moreover, the long-term stability of these new designs with regard to bone growth remains poorly understood. More research is required to understand the extent of potential improvements and to predict clinical outcomes.

## Data Availability

The review protocol, as well as the data generated and/or analyzed during the current study are available from the corresponding author on reasonable request.

## Abbreviations

AM: Additive manufacturing
CT: Computed tomography
DMLS: Direct metal laser sintering
DMP: Direct metal printing
EBM: Electron-beam melting
PRISMA: Preferred Reporting Items for Systematic Reviews and Meta-Analyses
QAQ: Quality assessment question
RVE: Representative volume element
SLM: Selective laser melting
SSI: Stress shielding increase
SSS: Stress shielding signal
STROBE: Strengthening the Reporting of Observational Studies in Epidemiology
THR: Total hip replacement
TKR: Total knee replacement
TSR: Total shoulder replacement
